# Are there differences in all-cause and coronary heart disease mortality between immigrants in Sweden and in their country of birth? A follow-up study of total populations

**DOI:** 10.1186/1471-2458-6-102

**Published:** 2006-04-21

**Authors:** Malin Gadd, Sven-Erik Johansson, Jan Sundquist, Per Wändell

**Affiliations:** 1 CeFAM/Center for Family and Community Medicine, Neurotec, Karolinska Institutet, Stockholm, Sweden

## Abstract

**Background:**

Mortality from cardiovascular diseases is higher among immigrants than native Swedes. It is not clear whether the high mortality persists from the country of birth or is a result of migration. The purpose of the present study was to analyse whether all-cause and coronary heart disease mortality differ between immigrants in Sweden and in the country of birth.

**Methods:**

Two cohorts including the total population from Swedish national registers and WHO were defined. All-cause and CHD mortality are presented as age-adjusted incidence rates and incidence density ratios (IDR) in eight immigrant groups in Sweden and in their country of birth. The data were analysed using Poisson regression.

**Results:**

The all-cause mortality risk was lower among seven of eight male immigrant groups (IDR 0.39–0.97) and among six of eight female immigrant groups (IDR 0.42–0.81) than in their country of birth. The CHD mortality risk was significantly lower in male immigrants from Norway (IDR = 0.84), Finland (IDR = 0.91), Germany (IDR = 0.84) and Hungary (IDR = 0.59) and among female immigrants from Germany (IDR = 0.66) and Hungary (IDR = 0.54) than in their country of birth. In contrast, there was a significantly higher CHD mortality risk in male immigrants from Southern Europe (IDR = 1.23) than in their country of birth.

**Conclusion:**

The all-cause mortality risk was lower in the majority of immigrant groups in Sweden than in their country of birth. The differences in CHD mortality risks were more complex. For countries with high CHD mortality, such as Finland and Hungary, the risk was lower among immigrants in Sweden than in their country of birth. For low-risk countries in South Europe, the risk was higher in immigrants in Sweden than in South Europe.

## Background

International migration increased considerably in the last decades of the 20th century because of people escaping war, poverty, and political, economic, and religious repression. In Sweden too, immigration has increased, and on 31 December 2003 12% of the total population was first-generation immigrants. Swedish society has become multicultural and multiethnic. The immigrant groups in this study mainly arrived in Sweden in 1940s–1970s and have today lived in Sweden for at least 30, sometimes up to 60 years. Most of the immigrant groups peaked during the 1950s and 1960s, except for Polish immigrants that arrived during the 1940s and immigrants from Chile during 1970s. The majority of the Polish, Hungarian, and Chilean immigrant groups arrived in Sweden as political refugees. The Hungarian immigrant group is well educated. The Polish group is more heterogeneous with both high and low education; those who arrived during the mid 20th century are well educated while those arriving today are less so. Both high and low education is seen among immigrants from Chile. The majority of the immigrants from Scandinavia and Germany arrived as high-skilled workforce immigrants, and the majority of the Southern European immigrants as low-skilled workers. This difference in education is also associated with differences in social status. Generally, the majority of the immigrants in Sweden live in urban areas.

Coronary heart disease (CHD) is one of the leading causes of death in the industrialized countries today and accounts for nearly a quarter of all deaths. In these countries with growing immigrant populations, distinct differences in mortality and morbidity from cardiovascular disease (CVD) and CHD is seen according to country of birth, with higher risks of CVD and CHD among the majority of immigrant groups [1-4], while other immigrants, particularly from southern Europe and other countries from the Organization for Economic Co-operation and Development (OECD), have lower risks of CVD [2,4,5].

The majority of male immigrant groups in Sweden have higher risks of coronary heart disease (CHD) than the Swedish-born majority population, even when level of education and employment status have been taken into account [1]. However, it has not been possible to draw any conclusions as to whether these high-risk levels persist from the country of birth or whether they are a result of migration or the acculturation process, which is a general weakness in most epidemiology research on migration. The rising flow of international migration during the last few decades has raised the number of first-generation immigrants in Sweden to about 12% (2004) of the total population. The purpose of the present study was therefore to analyse all-cause and CHD mortality in immigrant groups in Sweden and in their country of birth.

The incidence of death from CHD was studied in three defined cohorts of 45- to 68-year-old Japanese men in Japan, Hawaii, and California. The results showed a large increase in the incidence of CHD in the Japanese men who migrated to the US that was much more pronounced in California than in Hawaii [6,7]. However, there is a need to repeat such studies in a new context and in a new time. Unchanged CHD risk in immigrant groups in a country and in their country of birth might be interpreted as indicating that no changes in lifestyle or risk factors have occurred. Consequently, an increased CHD risk might be interpreted as deterioration in lifestyle or risk factors and significant acculturative stress. To investigate more closely the issue of mortality or morbidity changes as a result of migration, it is necessary to compare mortality or morbidity between immigrant groups and corresponding compatriots in their country of birth.

According to the hypothesis of the "healthy migrant effect", individuals who manage to migrate are healthier than those in their country of birth, [2,8-13], which may explain why some immigrant groups have lower morbidity or mortality than the population in the new country. On the other hand, several studies have shown that morbidity and mortality from CHD are higher among immigrants than in the majority population in the new country [1,2,5,14-21]. However, a higher risk of CHD than that of the majority population in the new country does not necessarily indicate a deterioration of health because the risk might still be lower than in the country of birth. There is a need to compare CHD risk in the new country with that in the country of birth. Only a few such studies have been performed [6,7].

Based on previous international and Swedish immigrant studies [1,2,5,14-21], we expected to find higher all-cause and CHD mortality risks among immigrants in Sweden than in their country of birth.

## Methods

The data in this study were taken from two large databases, the national database MigMed at the Karolinska Institute and data from WHO (table 1) [22]. The MigMed and WHO databases include annual data on entire populations. The MigMed is a result of linking several national registers such as the Population Register and the Cause-of-Death Register. Information about all-cause deaths and deaths from CHD by age and sex was obtained from both the databases. This study is therefore based on two separate cohorts aged 45–74 years: the WHO cohort in 1995, and the MigMed cohort, which includes eight years, from 1991 to 1998.

The different design of study periods between the databases was intended to include enough countries, and at the same time to include large enough populations, in the end, to give statistically reliable estimates. A limited number of countries were represented in the WHO database. In the MigMed, some immigrant groups were to small too give reliable number of deaths from CHD according to the size of the immigrant population. Many of the largest immigrant groups in Sweden were not represented in the WHO database, and those represented in the WHO database were small immigrant groups by Swedish standards. The only way to deal with this was to enlarge the immigrant groups in Sweden by expanding the study period. However, it was not possible to expand the study period in the WHO database in the same way since only data until 1995 were available when the study was performed. Also, it was not possible to bring the study forward since the MigMed database was established in 1991. By including data from both databases only during 1995, the size of the immigrant populations in Sweden would have been too small. We therefore expanded the study period of immigrants in Sweden to eight years, 1991–1998, with the median year of the study period in 1994/1995, estimated to be comparable to the WHO data for 1995.

### Variables

#### Outcome variables

All-cause mortality is defined as death from any cause. CHD mortality is defined according to the International Classification of Diseases, ICD9: 410–414; and ICD10: I20–I22, I24–I25.

#### Independent variables

*Age *was categorized into: 45–49, 50–54, 55–59, 60–64, 65–69, 70–74 years, excluding the oldest ages because of the small number of elderly immigrants in Sweden, and the youngest groups because death from CHD is rare in these age groups.

*Immigrants *were defined as persons born abroad and not having Swedish parents, i.e. first-generation immigrants.

In many of the immigrant groups in MigMed, the number of cases of CHD was too small to obtain reliable estimates, and these were consequently excluded from the study. Further, mortality data were lacking for some of the countries in the WHO database. Eight countries were finally selected: Denmark, Norway, Finland, Germany, Poland, Hungary, Southern Europe (France, Greece, Israel, Italy, Portugal, Spain) and Chile.

### Statistical analysis

Person-years at risk for the whole populations in the WHO cohort constituted the size of the population. In the MigMed cohort person-years at risk was obtained by summing up the population aged 45–74 years for each of the eight years. Age-adjusted all-cause and CHD mortality rates in the immigrant groups were calculated by indirect standardization with the Swedish population in the WHO database as reference [23]. A Poisson regression model was used to estimate the incidence density ratio (IDR) of all-cause and CHD mortality by using country of birth as reference group. The results are shown as incidence density ratios (IDR) with 95% confidence intervals (CI). The goodness of fit was tested and was satisfactory in 26/32 groups. The STATA software package was used in the statistical analyses [24].

The data from the WHO database were previously validated by WHO itself according to completeness and coverage. "Completeness refers to the proportion of all deaths that are registered in the population covered by the vital registration system for a country. Coverage is calculated by dividing the total deaths reported for a country-year from the vital registration system by the total deaths estimated by WHO for that year for the national population. The national population estimates used by WHO are those of the UN Population Division, 2002 revision. Best estimates of death rates by age and sex, adjusted for incompleteness, are applied to the national population data to obtain total estimated deaths. WHO estimated coverage for a Member State may be less than 100% due to incompleteness of registration, or to coverage of only some parts of the national population, or to differences between the vital registration population and the UN estimated de-facto population. Coverage higher than 100% might be due to the fact that some national registration systems might record both deaths of residents as well as non-resident deaths occurring in the country. In some cases, deaths of nationals dying abroad are also included. Furthermore, the UN estimated de-facto population might be lower than the vital registration population in some cases. Completeness is estimated at 100% in all groups studied, but the coverage varies from 92 to 108%, which are high and reliable results" (tables 3 and 4) [22].

## Results

The number of person-years at risk, the number of all-cause and CHD deaths, and age-adjusted all-cause and CHD mortality rates in different immigrant groups in Sweden and in their country of birth are shown in Table 1.

All-cause mortality rates ranged between 75 and 156 deaths per 10 000 person-years at risk among immigrant men in Sweden, and among immigrant women the range was from 42 to 83. The highest all-cause mortality rates were seen in immigrant men from Finland and immigrant women from Denmark, and the lowest rates in immigrant men and women from Chile. CHD mortality rates ranged between 13 and 46 deaths per 10 000 person-years at risk among immigrant men, and between 7 and 15 among immigrant women. The highest CHD mortality rates were seen in immigrant men from Finland and immigrant women from Denmark, and the lowest rates in immigrant men and women from Chile.

There are apparent mortality differences between countries. Among men the all-cause mortality rates ranged between 120 and 297 deaths per 10 000 person-years at risk, and among women they ranged between 64 and 141. The highest all-cause mortality rates were seen in men and women from Hungary, and the lowest rates in men from Sweden and women from Southern Europe. CHD mortality rates ranged between 18 and 64 in men and between 6 and 27 in women. The highest CHD mortality rates were seen in men and women from Hungary and the lowest rates in men and women from Southern Europe.

The all-cause relative mortality risk was significantly lower in Sweden than in the country of origin in immigrant men from Denmark (IDR = 0.8), Norway (IDR = 0.91), Germany (IDR = 0.65), Southern Europe (IDR = 0.66), Chile (IDR = 0.40), Poland (IDR = 0.5) and Hungary (IDR = 0.39), and in immigrant women from Denmark (IDR = 0.72), Germany (IDR = 0.71), Southern Europe (IDR = 0.81), Chile (IDR = 0.42), Poland (IDR = 0.66) and Hungary (IDR = 0.53). In the remaining groups, men from Finland, and women from Norway and Finland, the risks did not differ significantly from the country of birth (Table 2). The CHD mortality risk was also lower in men from Norway (IDR = 0.84), Finland (IDR = 0.91), Germany (IDR = 0.84), and Hungary (IDR = 0.59), and in immigrant women from Germany (IDR = 0.66), and Hungary (IDR = 0.54), than in their country of birth. Immigrant men from Southern Europe had a higher risk (IDR = 1.23; CI = 1.04–1.45) than in their country of birth. In the remaining groups, men from Denmark, Southern Europe, Chile, and Poland, and women from Denmark, Norway, Finland, Chile, and Poland, the risks did not differ significantly (Table 2).

## Discussion

The main finding of this study is the lower all-cause mortality risk among seven out of eight male immigrant groups and six out of eight female immigrant groups in Sweden than in their country of birth. Furthermore, the risk of CHD mortality was lower among immigrant men and women from Germany and Hungary and among immigrant men from Norway and Finland than in their country of birth. In contrast, a higher risk of CHD mortality was seen among immigrant men from Southern Europe who were settled in Sweden, than in their country of birth. The risk of CHD was equal to that in the country of birth in men from Denmark, Chile, and Poland, and in women from Denmark, Norway, Finland, Southern Europe, Chile, and Poland.

Based on results from an earlier study comprising immigrants aged 35–64 years, we expected to find higher all-cause mortality and CHD mortality risks among immigrants in Sweden than in their country of birth. In contrast, however, the present study showed higher all-cause mortality risks in their respective countries of birth than among immigrant groups in Sweden. There was a lower all-cause mortality risk in the majority of immigrant groups in Sweden than in their country of birth, in some cases remarkably lower. This might be interpreted as a decrease in all-cause mortality, but it might also be a sign of a selection bias, meaning that healthy migrants are more prone to emigrate, i.e. the "healthy migrant effect" [2,8,9,13] or, finally, to over-coverage. We assume that over-coverage is a consequence of re-emigration that is not reported to the authorities, meaning people who have left Sweden. Over-coverage is estimated at about 4–8 per cent of the non-Nordic immigrants [25,26], with the highest rates among immigrants originating from continents other than Europe. Over-coverage at this level probably does not affect the main results of this study.

Of the countries included in this study, Sweden had the lowest all-cause mortality and highest lifetime expectancy at birth, 77.3 years for men and 82.0 years for women. Increasing life expectancy shows a high negative correlation with increasing standardized mortality rates [27]. A relative mortality risk of 0.5 corresponds to an increased lifetime expectancy of around 5 years, i.e. the difference between men and women in most countries. Subsequently, the lower all-cause mortality risks in some immigrant groups seen in this study are by no means unrealistic. As an example, a study looking at neighbourhood areas in Chicago found that life expectancy for men ranged from 54 to 77 years [28]. Another example is that CHD mortality among 50-year-old Lithuanian men is four times higher than among 50-year-old Swedish men despite the similar standard risk factors, probably as a result of psychosocial risk factors [29].

There is very limited evidence from studies comparing CHD mortality between immigrants in a new country with corresponding compatriots in the country of birth. In one of the few studies conducted, a Japan-Hawaii-California gradient of increasing CHD mortality rates was found and explained by increasing distance from country of birth and increasing acculturation to a Westernized lifestyle [30]. These findings are in accordance with our results for the Southern European group, with higher CHD mortality among immigrants in Sweden than in their country of birth. One possible mechanism for lower rates of CHD may be "the Mediterranean diet" in Southern Europe and Japan. These food traditions are based on high consumption of vegetables, fruits, and fibre [31]. In contrast, the traditional diet in Sweden and the US is based on meat and dairy products. A comparison between Japan and the US in the Honolulu Heart Program study and Southern Europe and Sweden in our study shows high concordance. Despite recruitment of healthy Southern European workers by the Swedish government during the 1940s and 1950s, no sign of the "healthy migrant effect" was evident in this study. However, the effect was not found to be significant in female Southern European immigrants.

This study showed lower CHD mortality among Hungarian male and female immigrant groups in Sweden than in their country of birth. This might be a result of a "healthy migrant effect" or due to increased acculturation because of their lengths of stay in Sweden, and/or to improvement in the pattern of risk factors as a result of migration. Acculturation to the majority population will increase with increasing length of stay in Sweden. Immigrants from Poland and Chile who arrived in Sweden in the 1970s and 1980s may be less acculturated than Hungarian immigrants who came to Sweden in 1956 and who have been integrated into Swedish society for more than four decades. However, as there are no socio-economic or lifestyle factors in the WHO database, we could not account for any of these variables. These CHD risk factors differ between countries and populations and might change due to migration. For instance, in data from Statistics Sweden and WHO, the number of persons with a completed university education was greater among Hungarian immigrants in Sweden than among the population in Hungary, but less among immigrants from Southern Europe, OECD and the Nordic countries than in their country of birth.

It is likely that immigrants who acculturate to Swedish society and the majority population's lifestyle patterns will also have CHD and all-cause mortality risks that are closer to those of the majority population. Differences in lifestyle and risk factors may differ between the populations in this study. In a previous study, most immigrant men and women from Finland, Poland, Southern Europe, and Chile had higher risks of smoking and physical inactivity than Swedish men and women [14]. Finnish-born women also had a higher risk of diabetes than Swedish-born women. In addition, by migrating to Sweden, immigrants from Southern Europe leave a healthy "Mediterranean diet" based on fruit and vegetables for a Westernized diet based on dairy products and meat, and because of this the risk of CHD mortality might increase. An increased risk of CHD was also seen among Southern European men in this study.

### Limitations and strengths

Comparing data from different national databases introduces a selection bias. Although each individual country provides demographic and mortality data to WHO, the management routines in dealing with CHD mortality data, for example, might differ between countries. In this sense, the outcome "all-cause mortality" is ideal, as definitions are alike worldwide. Therefore, the data on all-cause mortality are probably most reliable. CHD is well defined according to ICD, but in practice, diagnosing CHD is probably associated with misclassifications. Mainly European countries are included in this study, and these countries usually have similar routines for dealing with, assessing, and compiling mortality data. The inclusion of primarily European immigrants has therefore minimized the problem of comparability and reliability. In addition, WHO has performed validations of the completeness and coverage of the WHO data. According to these validations, the completeness and coverage are acceptable for the countries included in this study.

Only the largest immigrant groups in Sweden, with large enough numbers of deaths to yield reliable estimates, could be included. By expanding the study period to eight years, we increased the number of groups that could be included. In the WHO statistics, based on whole populations, data from only one year were sufficient. Access to data in the WHO register was limited for a number of countries such as Turkey and countries in the Middle East, and this restricted the number of countries we could include in this study.

Data until 1997 in the WHO database, and data between 1991 and 1998 in MigMed, were accessible at the time when the study was performed. Choosing one single year to study would have made the MigMed cohort statistically too small. By expanding the years of study in the MigMed database, we maximized the significant results and thereby the number of countries that were possible to include. The maximized study period in MigMed was to use data from the beginning to the end, between 1991 and 1998. We estimated 1994–1995, the median years of this period, to be the best year to compare to in the WHO. Therefore, we chose to study 1995 in WHO database. But there are problems related to the comparison of aggregate data. The same individuals are included in several study periods. Subpopulations with high or low mortality might have a disproportionately high influence on the results.

There are problems in the regional grouping of countries, as with Southern Europe in this study. Southern Europe comprised six rather different countries: Greece, France, Israel, Italy, Portugal, and Spain. By fusing countries into a region, the specificity of each country disappears and positive or negative effects may be diluted. The characteristics, e.g. cultural, ethnic, political, and religious, of each country get lost by diluting the effect of the culture and religion. In this study, the all-cause mortality decreased in both sexes and the CHD mortality increased in men but remained unchanged in women. E.g., the increasing CHD mortality among South European immigrant men might conceal a decreased risk in some of the countries, but might also dilute the increased mortality that we describe.

Acculturation into a new country and culture has an influence on health through a change of lifestyle and risk factors for disease. The acculturation of immigrants into the new country increases with time. Therefore, the all-cause and CHD mortality among immigrants should, through time, approach the Swedish level, and recede from the level in the country of birth. Despite not having individual data on time living in Sweden, this aggregated information tells us that the immigrant groups in this study have usually lived 35–55 years in Sweden, a rather long time in relation to life expectancy. The majority of groups in this study are generally quite well acculturated into Swedish society and the differences in risk may reflect acculturation to Swedish culture, with the internationally low risks of all-cause and CHD mortality.

The fact that no data on lifestyle or other risk factors were available in the WHO or the MigMed databases is a limitation. Furthermore, no *socio-economic *variables are included in the WHO database. In order to make the models comparable, we therefore decided not to adjust the MigMed data for socio-economic variables.

Socio-economic status has an influence on health. In this study we compare mortality between immigrants in Sweden with the population in the country of birth. Individual data on socio-economic status were not available in the WHO database, and therefore were not adjusted for, but population-based figures were available. By looking at the population-based data we were able to see that the majority of immigrant groups analysed in this study, immigrants from Finland, the OECD, Poland, Southern Europe, and Chile, had a lower degree of education than the corresponding population in the country of birth, except for immigrants from Eastern Europe who had a higher degree of education than natives of East Europe (table 3). Figures of unemployment were not available from the WHO at the time of the study.

The differences in education between immigrants in Sweden and in their country of birth might have had an influence on our results in that the immigrants in Sweden should be less healthy, with higher mortality, than the population in the country of birth, partly explained by lower socio-economic status (education). But this was not the case; in general immigrants in Sweden had lower mortality than in the country of birth.

The limitations of this study are balanced by its strengths. For example, most registers in Sweden use a ten-digit personal identification number assigned to each resident, including refugees and immigrants staying in Sweden for more than three months. The ten-digit personal identification number makes it possible to link information from several different registers that include the entire population, as is done in the MigMed database. This has enabled us to create a very large sample: all Swedes aged 45–74 during the eight-year period. Furthermore, the information on mortality in MigMed is taken from the Cause-of-Death Register. In addition, WHO statistics are the most extensive and reliable international data available for assessment. Finally, the main strength of this study is the comparisons between the mortality in immigrant groups in Sweden and in their country of birth.

## Conclusion

Our findings of a remarkable lowering of all-cause mortality, and in some groups of CHD mortality, among immigrants in Sweden compared to that in their country of birth indicates that a "healthy migrant effect" might exist or that mortality rates have decreased due to migration. However, further studies are needed to determine the mechanism behind these results. Socio-economic status, lifestyle, risk factors, and stage of acculturation might explain the heterogeneity of our findings.

## Competing interests

The author(s) declare that they have no competing interests.

## Authors' contributions

MG conceived the idea and hypothesis, participated in the design and planning process, carried out the study by performing the statistical analysis, compiled the results, wrote the draft of the manuscript, and was active in the correspondence with journals. PW acted as the main supervisor, participated in the design and planning process, participated in the final writing of the manuscript. SEJ participated in the design and planning process, and was active in the final writing of the manuscript. JS acted as a second supervisor and participated in the final writing of the manuscript.

## Pre-publication history

The pre-publication history for this paper can be accessed here:



## Figures and Tables

**Table 1:** Person-years at risk, number of all-cause and CHD deaths, age-adjusted all-cause and CHD mortality rates, (per 10 000 person-years at risk) and life expectancy (years) in the different countries, among immigrant groups in Sweden and in their country of birth. The age of the study population is 45–74 years. The reference population is Swedes in the WHO database.

**Country**	**Person-years at risk**	**Number of all-cause deaths**	**Number of CHD deaths**	**All-cause mortality rate**	**CHD mortality rates**	**Life expectancy, years**
***WHO***^***1***^						

	*Men*					
	
Sweden	1 427 200^2^	17060^2^	5 096^2^	120^2^	36^2^	77.3^2^
Denmark	834 600	13 461	2 832	170	36	74.2
Norway	637 200	8779	2 486	138	39	75.7
Finland	799 500	12 171	3 769	163	51	73.7
Germany	13 252 200	204 018	44 610	157	35	74.3
Southern Europe	27 664 800	384 991	52 233	134	18	75.0
Chile	1 342 569	20 591	2 422	173	21	72.5
Poland	4 926 500	117 529	18 552	243	38	69.2
Hungary	1 536 618	45 557	9 735	297	64	66.3

	*Women*					
	
Sweden	1 480 900^2^	10 441^2^	1 756^2^	71^2^	12^2^	82.0^2^
Denmark	873 200	9 646	1 203	115	15	78.5
Norway	662 900	5 206	879	77	13	81.4
Finland	881 600	6 517	1 400	75	16	80.9
Germany	14 655 600	127 955	20 319	84	13	80.6
Southern Europe	30 446 500	203 332	19 052	64	6	81.4
Chile	1 509 217	13 315	1 179	99	9	79.5
Poland	5 876 400	67 921	7 772	114	13	77.7
Hungary	1 902 651	27 579	5 223	141	27	75.2

***MigMed***^1^						

	*Men*					
	
Sweden	10 210 682^3^	116 720^3^	34 612^3^	111^3^	33^3^	
Denmark	100 034	1 579	433	135	36	
Norway	71 954	1 059	283	125	33	
Finland	397 646	5 232	1 489	156	46	
Germany	85 310	833	235	103	29	
Southern Europe	75 264	551	136	92	22	
Chile	22 096	102	18	75	13	
Poland	37 341	425	114	121	32	
Hungary	40 190	450	143	121	38	

	*Women*					
	
Sweden	10 425 411^3^	71 218^3^	11 884^3^	68^3^	11^3^	
Denmark	86 269	799	135	83	14	
Norway	110 443	1 041	194	77	13	
Finland	549 085	3 611	681	76	15	
Germany	109 327	733	110	59	9	
Southern Europe	42 740	191	28	53	9	
Chile	24 479	83	12	42	7	
Poland	73450	481	85	77	14	
Hungary	29 544	212	40	76	15	

^1^*The data on immigrants in Sweden are taken from the database MigMed and data on country of birth from WHO*.

^2^*These figures are based on the all-cause deaths in the Swedish population from WHO *(*including immigrants*)

^3^*These figures are based only on Swedes from MigMed (excluding all immigrants)*.

**Table 2:** All-cause, and CHD mortality density ratio s (IDR), age-adjusted with 95% confidence interval (CI) in eight immigrant groups in Sweden and in their country of birth (IDR = 1) for the ages 45–74 years.

**IDR (CI)**	**CHD mortality**		**All-cause mortality**	
**Country**	Immigrants in Sweden	Country of birth	Immigrants in Sweden	Country of birth

	**Men**			
	
Denmark	1.01 (0.91 – 1.12)	1	0.80 (0.76 – 0.84)	1
Norway	0.84 (0.75 – 0.96)	1	0.91 (0.85 – 0.97)	1
Finland	0.91 (0.86 – 0.97)	1	0.97 (0.94 – 1.00)	1
Germany	0.84 (0.74 – 0.96)	1	0.65 (0.61 – 0.70)	1
Southern Europe	1.23 (1.04 – 1.45)	1	0.66 (0.60 – 0.72)	1
Chile	0.65 (0.41 – 1.04)	1	0.40 (0.33 – 0.49)	1
Poland	0.86 (0.71 – 1.03)	1	0.50 (0.45 – 0.55)	1
Hungary	0.59 (0.50 – 0.69)	1	0.39 (0.36 – 0.43)	1
	
	**Women**			
	
Denmark	0.93 (0.78 – 1.11)	1	0.72 (0.67 – 0.77)	1
Norway	1.01 (0.87 – 1.18)	1	0.98 (0.91 – 1.04)	1
Finland	0.95 (0.87 – 1.05)	1	1.01 (0.97 – 1.05)	1
Germany	0.66 (0.54 – 0.79)	1	0.71 (0.66 – 0.76)	1
Southern Europe	1.37 (0.94 – 1.98)	1	0.81 (0.70 – 0.93)	1
Chile	0.70 (0.39 – 1.23)	1	0.42 (0.34 – 0.52)	1
Poland	1.05 (0.88 – 1.30)	1	0.66 (0.60 – 0.72)	1
Hungary	0.54 (0.39 – 0.73)	1	0.53 (0.46 – 0.61)	1
